# Is heat stress a growing problem for dairy cattle husbandry in the temperate regions? A case study of Baden-Württemberg in Germany

**DOI:** 10.1093/jas/skae287

**Published:** 2024-09-23

**Authors:** Miguel António Leandro, Joana Stock, Jörn Bennewitz, Mizeck G G Chagunda

**Affiliations:** University of Hohenheim, Department of Animal Breeding and Husbandry in the Tropics and Subtropics, Stuttgart, Germany; University of Hohenheim, Department of Animal Breeding and Husbandry in the Tropics and Subtropics, Stuttgart, Germany; University of Hohenheim, Department of Animal Breeding and Genetics, Stuttgart, Germany; University of Hohenheim, Department of Animal Breeding and Husbandry in the Tropics and Subtropics, Stuttgart, Germany; Centre for Tropical Livestock Genetics and Health (CTLGH), The University of Edinburgh, Easter Bush Campus, Midlothian EH25 9RG, United Kingdom

**Keywords:** ambient temperature, heat stress, microclimate, relative humidity, Temperature–Humidity Index

## Abstract

Heat stress with measurable effects in dairy cattle is a growing concern in temperate regions. Heat stress in temperate regions differs between environments with different geophysical characteristics. Microclimates specific to each environment were found to greatly impact at what level heat stress occurs and will occur in the future. The landlocked state of Baden-Württemberg, Germany, provides several different environments, hence, a good case-study. Temperature–Humidity Index (**THI**) from 17 weather stations for the years 2003 to 2022 was calculated and milking yields from 22 farms for the years 2017 to 2022 were collected. The occurrences and evolving patterns of heat stress were analyzed with the use of a THI, and the effect of heat stress on milk yield was analyzed based on milking records from Automated Milking Systems. Daily average THI was calculated using hourly readings of relative humidity and ambient temperature, disregarding solar radiation and wind, as all animals were permanently stabled. Based on studies conducted in Baden-Württemberg and neighboring regions, cited ahead in the section of THI, THI = 60 was the threshold for heat stress occurrence. Findings show that the heat stress period varied between stations from 64 to 120 d with THI ≥ 60 in a year. This aligns with yearly and summer averages, also steadily increasing from May to September. The length of the heat stress period was found to increase 1 extra day every year. Extreme weather events such as heat waves did not increase the heat stress period of that year in length but increased the average THI. Milk yield was found to be significantly (α = 0.05) different between counties grouped into different zones according to heat stress severity and rate of increase in daily average THI. Future attempts at managing heat stress on dairy cattle farms in the temperate regions should account for microclimate, as geographical proximity does not mean that the increase in heat stress severity will be the same in the 2 neighboring areas.

## Teaser

Temperate regions can be mapped into different heat stress zones where milk yield is significantly decreased to different levels with an overall tendency for worsening the existing situation.

## Introduction

Stress in dairy cattle is a major cause of performance and production losses with a detrimental effect on the animal’s health (Tao et al., 2020). The term stress is used to define any condition considered detrimental to the well-being and normal performance of an animal (Hafez, 1968). Many different sources of stress in dairy cattle are associated with a significant lack of growth and milk production, such as climatic-, nutritional-, and social stress, as well as internal stress caused by disease (Stott, 1981). When husbandry does not account for environmental stressors and mitigation strategies are not applied, stressor effects increase (Munksgaard and Løvendahl, 1993), resulting in a higher risk of lameness (Rushen, 2001), mastitis (Pragna et al., 2016), hyperthermia, reduced fertility (Sammad et al., 2020) and other disorders. Environmental stressors encompass those induced by climate (i.e., extreme temperatures and humidity), nutrition (i.e., improper feed or feed quality), and management (i.e., transport and housing conditions) (Bova et al., 2014). Although stress can be classified into distinct categories, those do not occur independently from each other. For example, heat stress can cause dysregulation of an animal’s immune system, and simultaneously provide good conditions for the festering of disease. This can lead to physiological stress, which can lead to the inability to feed and participate naturally in the herd, leading to nutritional and social stress (Becker et al., 2020). Tests have shown that after 20 d of lying deprivation or of social isolation, growth hormone levels were highly reduced (Munksgaard and Løvendahl, 1993). Similarly, heat stress has been found to greatly reduce fertility and milk production (Grinter et al., 2023) and stress generated by improper feeding has also been shown to be positively correlated with reduced milk production (Bugueiro et al., 2021). Furthermore, a focus on breeding for high milk production results so that when feed intake is lowered, the animal’s energy reserves are greatly reduced and cannot be used to cope with stress (Rushen, 2001; Sammad et al., 2020).

Heat stress in an animal, that is, the state of stress caused by hyperthermia and failure to maintain thermoneutrality, affects animal production at several levels (André et al., 2011). Heat stress is an increasingly recurring issue as a consequence of climate change patterns, namely the reported average increase of global temperature by 1.5 °C, and the occurrence of extreme events such as heatwaves (Wankar et al., 2021). The metabolic heat load of a lactating cow increases proportionally with the milk production and requires dissipation, increasing the animal’s sensitivity to increases in ambient temperature and relative humidity (Spiers et al., 2004; André et al., 2011). Studies have shown that high-producing dairy cows are at the highest risk of suffering from heat stress (Bianca, 1965). Cows suffering from heat stress show at the early stages an elevated respiratory rate, succeeded by symptoms of reduced feed intake, sweating, severe heat stress, and lethargy (Spiers et al., 2004; Grinter et al., 2023). Heat stress has been shown to reduce the release of thyroidal hormones related to metabolic heat production, leading to a reduction in metabolic activity (André et al., 2011; Becker et al., 2020; Tao et al., 2020; Grinter et al., 2023). West (2003) compiled data from different studies where it was found that an increase of the average temperature from 26 to 29 °C, at average relative humidity in the test region (approximately 40%), only reduced the milk production in Holstein, Jersey, and Brown Swiss dairy cows by 3%, 7%, and 2% from the average, respectively. With the same temperature increase, but at 90% relative humidity, the average yield was decreased by 31%, 25%, and 17%, respectively. The author also stated that the decline in milk yield is, to some extent, mitigated with 3 to 6 h/d of temperatures under 21 °C. Hossain et al. (2022) found that when the Temperature–Humidity Index (**THI**) value increased by 17%, the average milk yield decreased by 24.4%, the protein content was reduced by 15.2%, the fat content by 14.5%, and the ash content was increased by 15.3%. The increase in ash content most likely meant only a higher percentage of minerals in the dry-matter of the milk, this might have been due to a lesser dilution effect by the lack of protein and fat. However, an increase in udder oxidative stress, either by mastitis or metritis could also cause the release of minerals into the milk (Ayemele et al., 2021).

In the temperate regions, heat stress has been described as inhibiting the success of decades of breeding for high-producing animals (Kadzere et al., 2002). The underlying biological mechanism is that high production has been achieved through higher feed intake, resulting in much higher metabolic heat which, in turn, is not dissipated when ambient temperature and relative humidity are high, leading to hyperthermia (Kadzere et al., 2002; Spiers et al., 2004; André et al., 2011). So far, mitigation in the temperate regions has relied on strategies, such as forced ventilation, air recirculation, sprinklers, and misters, adapting feeding times, feed additives and breeding (Ji et al., 2020), and on the positive effects of nights with temperatures below 21 °C (West, 2003; Hammami et al., 2013). Strong cooling systems and full protection from the outside environment (ambient temperature, relative humidity, wind, and solar radiation) are more prevalent in the tropics and subtropics, where the effects of heat stress on milk production are more pronounced, as opposed to the temperate regions, where unwalled stables and forced ventilation are more common (Grinter et al., 2023). Studies on voluntary cooling methods (Grinter et al., 2023), breeding, and other heat stress management methods have been continuously performed in the South of the United States for the past several decades but the effects of heat stress are now detected as far North as Canada (Polsky and Von Keyserlingk, 2017; Ouellet et al., 2019; Campos et al., 2022). Also, the number of days per year in the temperate regions with a continuous period at temperatures below 21 °C is decreasing (Silanikove and Koluman (Darcan), 2015) and heat stress is now a growing concern in the temperate regions as it has long been in the tropics and subtropics. The potential and actual effects of climate change, namely “global warming”, have been theorized and analyzed since the early 1990s. Using models available at the time, Klinedinst et al. (1993) predicted up to 35% decreases in fertility and milk production in the southern United States and Southern Europe, and up to 20% in the temperate regions of those same continents over 20 following years. While drastic reductions in milk yield and fertility occurred, the latter was found to be driven not only by an increase in temperature but also by a production-driven breeding program (Sammad et al., 2020).

Heat stress can be computed with the use of several environmental factors: ambient temperature, relative humidity and precipitation, solar radiation, and wind (Bohmanova et al., 2007). It is, however, more common to compute it only as a function of ambient temperature and relative humidity (Brügemann et al., 2012). Heat stress is then computed as a THI accounting for those parameters (Erbe et al., 2022). Stress is worse at higher ambient temperatures and higher relative humidity, as high relative humidity reduces the ability to remove heat through the evaporation of sweat and in the lungs (Bohmanova et al., 2007; Brügemann et al., 2012; Erbe et al., 2022). When the THI value surpasses a threshold specific to each species or breed, then the occurrence of heat stress is assumed with higher values indicating a more severe heat stress situation (Erbe et al., 2022). Furthermore, individual animals can show different levels of heat stress tolerance, a parameter commonly assessed in breeding against that stressor. While heat stress and its detrimental effects on milk production have been widely detected within the temperate regions (Grinter et al., 2023), the severity of heat stress within the temperate regions is yet to be determined. Furthermore, identifying a trend of how heat stress in the temperate regions is evolving would inform future scenarios and potential mitigation strategies. The aim of this study was to determine whether, in the past 20 yr, heat stress occurrence in the temperate regions has increased. Secondly, whether the severity of heat stress in terms of average THI has increased. Thirdly, if both previous hypotheses are confirmed, were the findings equally applicable to different environments within the temperate regions. Lastly, whether milk production in the temperate regions has been noticeably affected by the occurrence of heat stress.

To reach these research aims, milk production in relation to weather data within the German federal state of Baden-Württemberg was used as a case study. This region is landlocked, varying only 2 degrees in latitude (47.5°N to 49.5°N), and 3° in longitude (7.5°E to 10.5°E). Present at both East and West borders are flatlands crossed by rivers, mountains at the North border, and the Lake of Constance and the Alps at the South border. The territory is mainly composed of hills and mountains ranging from 85 to 1.846 m above sea level and the river valleys separating them, generating distinct wet and dry areas. Its landlocked position excludes the influence that large bodies of water have on climate, namely relative humidity is not affected, with the exception of the areas surrounding the Lake of Constance. The prevailing winds at this latitude are westerlies, but a strong presence of descending winds from the mountains to the North and South occurs as well and is detected in the North and South halves of the region, respectively (Ministerium für Umwelt, Klima und Energiewirtschaft Baden-Württemberg, 2014; Landesanstalt für Umwelt Baden-Württemberg, 2023). Local topography, the presence of bodies of water, and vegetation type (forests, tundra, shrublands, among many others) alter climate beyond the overarching climate pattern for that latitude. Such local climate patterns are referred to as microclimates (Zellweger et al., 2019). In the federal state of Baden-Württemberg, different microclimates of the temperate regions are present within a small latitude variation, minimizing differences in the impact of climate change due to varying latitudes. Furthermore, milk production is present in these different environments, so, heat stress differences between the environments and their effects on milk production can be compared, and the effect of microclimates and climate change can be evaluated.

## Materials and Methods

The methodology in this study involving animals adhered to the guidelines established by the University of Hohenheim, as approved by the German Research Society (Deutsche Forschungsgemeinschaft). All animals were housed on farms under the supervision and guidelines of the Ministry for Nutrition, Rural Area and Consumer Protection of Baden-Württemberg (Ministerium für Ernährung, Ländlichen Raum und Verbraucherschutz Baden-Württemberg).

### Case study and dataset

The test area used was the Federal State of Baden-Württemberg within the Federal Republic of Germany where data from 17 weather stations, covering 23 counties where dairy cattle production is present, was collected for the years from 2003 to 2022. As Dallago et al. (2021) reported, in Germany in the years 2010 to 2020, the culling age for dairy Holstein and Jersey cows was 3.24 yr of production, following 22 to 25 mo prior to first calving. The period of 20 yr from 2003 to 2022 was chosen as it accounted for 4 generations of animals. Furthermore, given the small latitude and longitude variation of the test region, climate change effects were expected to be similar by region, and most variation was expected to depend on microclimates. As described in the study of Gwinner (2011), the State of Baden-Württemberg is landlocked and blocked geographically from any saltwater mass, the Mediterranean is far away across the Alps and the North Sea is far away to the North. No noticeable weather distortion typical of proximity to the sea was expected. Albeit a small landmass, the State of Baden-Württemberg encompasses a South and North border with mountain ranges, the Alps and the Bergstraße, respectively. The West border is dominated by the Rhein River, and to the East most of the border is on flat land, except for the Danube valley and its affluents, primarily the Iller River. Longitudinally (North-South), Baden-Württemberg is crossed by the Black Forest’s hills and mountains, and diagonally by the Swabian Alps. These hills and mountains capture the greater amount of precipitation, the remaining flatlands have low precipitation averages among them and are fed mostly by the rivers crossing them. The prevailing winds at the latitudes from 47.5°N to 49.5°N are westerlies and, in this region, due to the North and South mountainous borders, dry descending wind is also present, averaging the major winds at a variation between South-West and North-West and the internal topography produces some sheltered regions with different wind patterns (Landesanstalt für Umwelt Baden-Württemberg, 2023). Dairy production in Baden-Württemberg is distributed in an arch, from the North-West close to the city of Mannheim, then East over the Swabian Alps, and into the Allgäu hills on the South-East by the Lake of Constance. Dairy production is also present in the Black Forest (Statistisches Landesamt Baden-Württemberg, 2011). In the West of Baden-Württemberg, following the banks of the River Rhine, substantial dairy production is absent (Lassen et al., 2008). Meat production in this western part of Baden-Württemberg is common, as evidenced by a majority of beef breeds (Seitz, 2010).

In Table 1, the counties chosen and their referring weather station number, as well as the geophysical characteristics of each county, are presented. The counties were chosen according to the farms participating in ongoing heat stress research in dairy cattle throughout the state of Baden-Württemberg. Station numbers 6, 8, 10, 13, and 15 are located in valleys; station numbers 3, 4, 7, 12, and 16 are located in hills; and station numbers 1, 2, 5, 9, 11, 14, and 17 are located in mountain ranges.

**Table 1.** Weather station numbers, corresponding counties and regions, and their geophysical description (altitude and topography)

Station	County	Region	Station altitude (m)	Topography	Wind direction
1	Stödtlen, Unterschneidheim, Aalen	Swabian Alps	463	Mountains—north side	South-East
2	Hüfingen, Tuningen	Swabian Alps	673	Mountains—west side	South-West
3	Murrhardt	Hohenlohe–Haller–Ebene	489	Plateau	East
4	Vellberg, Schwäbisch Hall	Hohenlohe–Haller–Ebene	432	Plateau	South-East
5	Nellingen	Swabian Alps	687	Mountain top	South-East
6	Balzheim	Iller Valley	539	High river valley	South-West
7	Aichstetten	Allgäu	614	Hills	South-West
8	Ravensburg	Mittleres Schussental	441	Valley	South-West
9	Riedlingen	Swabian Alps	534	Mountains—south side	South-West
10	Freiberg	Neckar Valley	251	River valley	West
11	Inzigkofen	Swabian Alps	581	Mountains—south side	South-West
12	Achberg	Allgäu	508	Hills	South-West
13	Marbach am Neckar	Neckar Valley	314	River valley	West
14	Weinheim	Bergstraße	98	Foothills—south side	North
15	Mulfingen	Jagst Valley	385	River valley	East
16	Kißlegg, Argenbühl	Allgäu	672	Hills	South-West
17	Balingen, Horb am Neckar	South Black Forest	619	Mountains—east side	South-West

**Figure 1.**
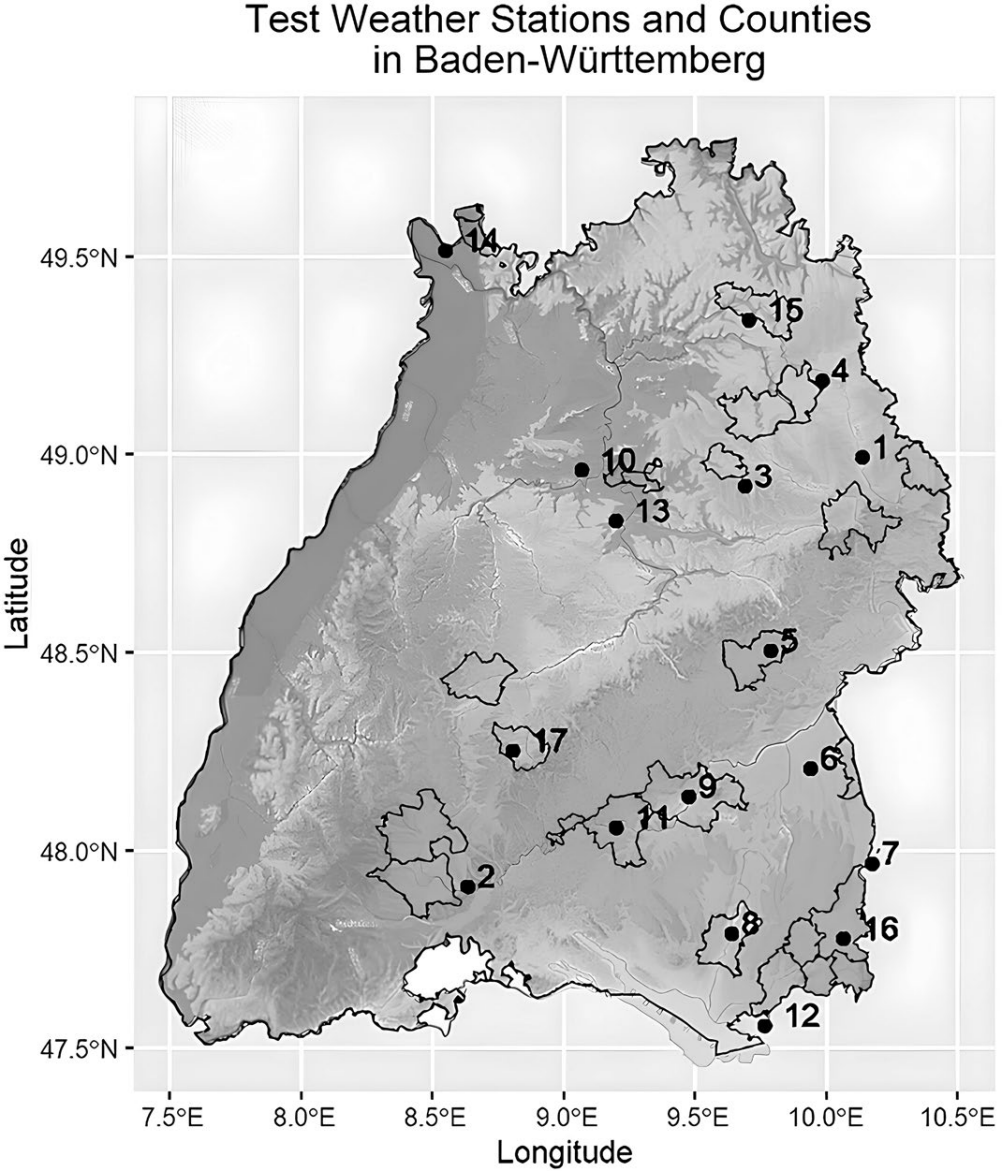
Map of the test weather stations and its corresponding counties superimposed over a topographic map of Baden-Württemberg, where lighter colors indicate higher altitudes and darker colors indicating lower altitudes within an altitude variation of 87 to 1493 m (Landesamt für Geoinformation und Landentwicklung, 2021).

In Figure 1, the same counties and stations as in Table 1 are shown within the map of Baden-Württemberg. The absence of dairy farming in the West is noteworthy. Stations 1, 2, 3, 4, 6, 7, 10, 12, 13, 14, and 15 are 7 to 20 km outside the counties for which they provide data, but are, however, the closest weather stations within the network of weather stations in Baden-Württemberg. Stations 1, 2, 4, 16, and 17 cover more than one county studied (Table 1). Stations 7 and 12 are the closest to their counties (Aichstetten and Achberg, respectively) but find themselves within the neighboring Federal State of Bavaria, 14 and 17 km away, respectively.

### Data collection

From the 17 weather stations, data was provided for daily precipitation (mm), daily snow depth (cm), hourly ambient temperature (°C), and hourly relative humidity (%), for the period between January 1, 2003, and December 31, 2022. For the counties with stations that ceased functioning during this 20-yr period, the substitute closest stations, as provided by the German Weather Office (*Deutscher Wetterdienst*) were used for the whole period, disregarding the ceasing stations. As such, all stations used produced data for the whole period uninterrupted. The data was provided pre-cleansed, purged of errors such as non-existent relative humidity readings or ambient temperature not changing throughout the day. Each station was identified per county and using coordinates for geolocation.

Milk yield data from the Automated Milking Systems of 22 dairy farms located in the counties covered by the weather stations was also provided. The data was provided anonymized and as such, farm-specific parameters such as management, feeding, and type of barn were unknown. This data included all individual milkings between September 1, 2017, and December 31, 2022.

### Temperature–Humidity Index

The THI was calculated according to the equation by the National Research Council, USA (1971):


$$\begin{array}{lll} THI= & \;31.9+0.45\times {T\circ }_{F}+0.55\times \left(\frac{RH\%}{100}\right)\; & \times {T\circ }_{F}-31.9\times \left(\frac{RH\%}{100}\right) \end{array}$$

where THI is the Temperature–Humidity Index, ${T\circ }_{F}$ is the ambient temperature in Fahrenheit, RH% is the relative humidity in percent, 31.9 is the intercept of the THI model of multiple linear regressions, 0.45 is the slope of the main effect “Ambient Temperature”, 0.55 is the slope alteration factor of the interaction between the main effects “Ambient Temperature” and “Relative Humidity”, and −31.9 is the slope of the main effect “Relative Humidity.

As this formula has been designed for the imputing of temperature in the Fahrenheit scale, an adapted version adjoining the conversion to the Celsius scale was used (Mader et al., 2005; Bohmanova et al., 2007; Erbe et al., 2022). In this adapted formula, the term ${T\circ }_{F}=1.8\times {T\circ }_{C}+32$ has been substituted to all temperature parameters, resulting in the following version:


$$\begin{array}{lll} THI=\; & 46.3+0.81\times {T\circ }_{C}+0.99\times \left(\frac{RH\%}{100}\right)\; & \times {T\circ }_{C}-14.3\times \left(\frac{RH\%}{100}\right) \end{array}$$

where THI is the Temperature–Humidity Index, ${T\circ }_{C}$ is the ambient temperature in Celsius, and RH% is the relative humidity in percent.

With the use of this formula, the threshold above which milk production has been found to be substantially reduced in Holstein Frisian cows in Germany was that of THI = 60 (Bohmanova et al., 2007; Brügemann et al., 2012; Zare-Tamami et al., 2018; Erbe et al., 2022). The same threshold was assumed for this study.

Hourly THI values were calculated from the 24 hourly readings of ambient temperature and relative humidity. The hourly THI values were then averaged as daily THI values for each weather station for the 20 yr period. For the analysis of the occurrence of heat stress, the heat stress period, that is, the days in a year with average THI above a threshold for physiological stress, was calculated, as follows. As THI ≥ 60 has been reported to induce physiological stress responses (Bohmanova et al., 2007; Brügemann et al., 2012; Zare-Tamami et al., 2018; Erbe et al., 2022) and symptomatic animals have been found within one day of heat stress occurring (Bernabucci et al., 2014), the total annual number of days with an average THI value equal or above 60 per station (Days_ta_) was counted and compared for its change throughout the 20 yr period. The main heat stress period always occurs in the summer months, however, during Spring and Autumn, periods of heat stress can occur. As effects on production parameters, namely milk production and estrus detection, have been reported by other studies to appear after 3 to 5 d of exposure to heat stress (Cavestany et al., 1985; De Rensis et al., 2015), the count of heat stress days was then conditioned to the number of days with THI equal or above 60 when 5 or more consecutive days occurred (Days_c_).

The severity of heat stress was assessed as the average yearly THI and as the average summer THI. For this analysis, the daily average of THI per station and summer month (June, July, and August) was calculated and compared throughout the 20 yr. A similar method for the evaluation of heat stress throughout several years using THI values calculated from data provided by weather stations was described in Hossain et al. (2022). The same analysis was performed for calculating the average yearly THI. For the analysis of the geographical distribution of heat stress, the rate of change in summer THI and the average yearly THI were compared to distinguish heat stress zones through means of K-Means cluster analysis (Hartigan and Wong, 1979) using RStudio (Posit team, 2023. RStudio: Integrated Development Environment for R. Posit Software, PBC, Boston, MA. URL http://www.posit.co/).

### Heat stress effect on milk production

In this study, the average milk yield per milking in kilogram for each month and year combination was calculated for each farm. This data was then compared to the results of the classification of the counties into heat stress zones, based on the analysis of the THI, and the average milk yield difference between heat stress zones was tested for significance.

### Statistical analysis

For the analysis of the change in the duration of the total heat stress period in days per year (Days_a_) and the number of heat stress days per year counted when 5 or more consecutive days occurred (Days_c_), a generalized linear mixed model with the log link-function was fitted, adjusting for overdispersion:


$$\begin{array}{l} y_{ij}=\log\left(\mu +S_{i}+\beta x_{ij}+\delta_{i}x_{ij}+Y_{ij}+e_{ij}\right) \end{array}$$

where *y*_*ij*_ is the number of days (Days_a_ or Days_c_, a Poisson distribution), µ is the common intercept, *S*_*i*_ is the effect of the *i*th weather station (fixed), β is the common regression slope, *x*_*ij*_ is the time (“Year” as a quantitative factor) corresponding to *y*_*ij*_ (fixed), δ_*i*_ is the deviation from the common slope for the *i*th weather station, *Y*_*ij*_ is the time (“Year” as a qualitative factor, random), and *e*_*ij*_ is the error of the observed count, ~N(0, $I\sigma_{e}^{2}$).

The fitted generalized linear mixed model accounting for Poisson distribution and adjusting for overdispersion was chosen as the data is in the form of a time series, where throughout the studied period, the total number of heat stress days was counted for each weather station in successive years and the number days is always a whole positive number with a limit of 365 d/yr. The variable “Time” was accounted for as a quantitative variable, every year in succession, and as a qualitative variable (denoted as “Year”) for the lack-of-fit test, in which each year is a separate class, yielding a distinct number of heat stress days per year.

For the analysis of the average summer THI, also a linear mixed model, accounting for repeated measurements was fitted:


$$\begin{array}{lll} y_{ijk}= & \;\mu +S_{i}+\beta x_{ij}+\delta x_{ij}+m_{k}+{\left(sm\right)}_{ik}\; & +Y_{ij}+(sY_{ij})+{\left(Ym\right)}_{ijk}+e_{ijk} \end{array}$$

where *y*_*ijk*_ is the monthly average THI for the *k*th summer month, *j*th year, and *i*th weather station, µ is the common intercept, *S*_*i*_ is the effect of the *i*th weather station (fixed), β is the common regression slope, *x*_*ij*_ is the time corresponding to *y*_*ij*_ (fixed), *δ*_*i*_ is the deviation from the common slope for the *i*th weather station, *m*_*k*_ is the effect of *k*th month, (*sm*)_*ik*_ is the interaction effect of the *i*th weather station and the *k*th month, *Y*_*ij*_ is the time (yr) as a qualitative factor (random), (*sY*)_*ij*_ is the interaction effect of the *i*th weather station and the *ij*th year, (*Ym*)_*ijk*_ is the interaction effect of the *ij*th year and the *k*th month, and *e*_*ijk*_ is the error for the observed THI measurement *y*_*ijk*_, $∼(0,\sigma_{e}^{2}\rho^{\left|j1-j2\right|})$.

As with the analysis of the heat stress period in days per year, the average summer THI data is a time series, so a linear mixed model was chosen. “Time” was also accounted for as both a quantitative and qualitative variable, with the addition that the variable “Month”, nested in the year it belongs to, was also included in the model. As such, the interaction between “Month” and “Station”, “Year” and “Station”, as well as “Year” and “Month” were all included in the model. An auto-regressive variance–covariance structure was modeled ($\sigma_{e}^{2}\rho^{\left|i-j\right|}$, where ρ is the autocorrelation parameter and *j* is the index of time order), as each month is necessarily more correlated to the one directly before and the one directly after than any other further apart, and it accounts for the regularity of measurements (one per month) within the “Time” axis. This same model was used for the analysis of the Yearly Average THI with the omission of the month-specific terms.

For the analysis of the effect of heat stress on the average milk yield per milking, expressed as the classification of each farm into the heat stress zone they are in, the following linear mixed model was fitted:


$$\begin{array}{l} y_{ijk}=\mu +Z_{i}+F_{jk}+D_{j}+{\left(ZD\right)}_{ij}+e_{ijk} \end{array}$$

where *y*_*ijk*_ is the average milk yield per milking of the *i*th heat stress zone, *j*th date (month and year combination), and *k*th farm nested within the *i*th heat stress zone, µ is the common intercept, *Z*_*i*_ is the main effect of the *i*th heat stress zone (fixed), *F*_*jk*_ is the effect of the *k*th farm, nested within the *i*th heat stress zone, *D*_*j*_ is the main effect of the *j*th date, (*ZD*)_*ij*_ is the interaction between the *i*th heat stress zone and the *j*th date, and *e*_*ijk*_ is the error for the observed milk yield y_ijk_, $∼(0,\sigma_{e}^{2}\rho^{\left|j1-j2\right|})$.

As with the data in the analysis of the heat stress period in days per year and average summer THI, the data of the average milk yield per milking is in the form of a time series, and so a linear mixed model was fitted. The auto-regressive variance–covariance structure was chosen, as 2 measurements close in time are more correlated than 2 measurements farther apart.

Statistical analysis was performed using PROC GLIMMIX for the analysis of the number of heat stress days per year and PROC MIXED for the analysis of the average summer THI and the effect of heat stress on the average milk yield per milking in SAS version 9.4 (SAS Institute Inc.).

## Results

### Heat stress period in number of days per year

The results of the analysis on the total number of days per year with an average THI value above 60 and the number of aggregated days per year when a minimum of 5 consecutive days with an average THI value above 60 occur are shown in Table 2.

**Table 2.** Least Squares Means (LSMEANS) of the number of heat stress days per year with THI ≥ 60 for each region and each year from 2003 to 2022, in total count (Days_ta_) and conditional count when a minimum of 5 consecutive heat stress days occurred (Days_c_). THI was calculated using average daily ambient temperature and relative humidity

Region	Average days THI ≥ 60 per year ± standard error (Days_ta_)	Average days THI ≥ 60 for at least 5 consecutive days, per year ± standard error (Days_c_)
Bergstraße	119.8 ± 3.4	106.5 ± 4.1
Neckar Valley—North	112.4 ± 3.3	96.5 ± 3.9
Neckar Valley—South	107.3 ± 3.3	94.2 ± 3.8
Schussental	94.6 ± 3.1	80.6 ± 3.5
Allgäu—South	92.3 ± 3.0	76.2 ± 3.4
Jagst Valley	91.0 ± 3.0	72.9 ± 3.4
Swabian Alps—Northeast	82.7 ± 2.9	65.9 ± 3.2
Hohenlohe–Haller–Ebene—Northeast	82.1 ± 3.2	64.2 ± 3.5
Iller Valley	81.9 ± 2.8	66.0 ± 3.2
Allgäu—North	75.2 ± 2.7	59.3 ± 3.0
South Black Forest	74.4 ± 3.2	54.4 ± 3.4
Hohenlohe–Haller–Ebene—Southwest	73.9 ± 3.4	56.9 ± 3.7
Swabian Alps—Southwest	73.3 ± 2.8	59.5 ± 3.1
Swabian Alps—Center	72.5 ± 3.2	57.1 ± 3.5
Swabian Alps—Center	72.2 ± 2.7	57.8 ± 3.0
Allgäu—Center	67.8 ± 2.9	51.9 ± 3.1
Swabian Alps—Center	64.4 ± 2.5	48.7 ± 2.7

The different regions in Baden-Württemberg had distinct differences in incidence of heat stress. The northern regions showed a higher number of heat stress days per year than the southern, and the eastern regions showed a higher number of heat stress days per year than the western. The highest values in heat stress days per year, ordered North to South and East to West were as follows: the Bergstraße region at 119.8, the Neckar Valley at 112.4, the Schussental and Jagst Valley at 94.6, the Swabian Alps, the Black Forest and the Allgäu ranging from 64.4 to 92.3. The Northeastern Swabian Alps showed a higher incidence of heat stress than the South and Center, with 82.7 against 72.2 d/yr, respectively. The Allgäu region showed no pattern with 3 stations yielding 92.3, 75.2, and 67.8 d/yr. The 2 stations in the Hohenlohe–Haller Plateau yielded 82.1 and 73.9 d/yr, and the station in the Iller Valley yielded 81.9 d/yr. The pattern of the highest number of heat stress days in the north compared to the south is clearly visible, confirming the absence of a latitude effect, where heat stress would be expected to occur the most in the south.

**Figure 2.**
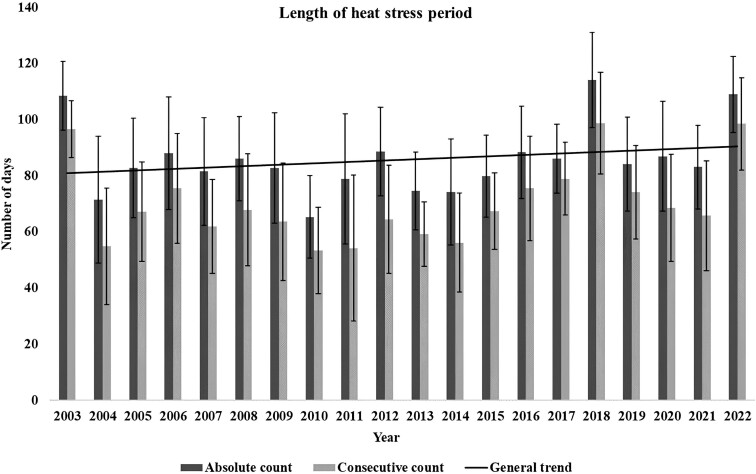
Comparison between the heat stress period length of the total annual count (all days per year with average THI ≥ 60) and conditional count (only days within periods of 5 or more consecutive days with average THI ≥ 60 were counted), averaged across all test regions. Error bars indicate standard deviation.

The counting of heat stress days per year when accounting only for periods with a minimum of 5 consecutive days yielded between 13 and 20 heat stress days less per year than the total annual count. Nevertheless, the ranking remained mostly unchanged and changes visible in Table 2 can all be explained by the standard error. Statistical analysis yielded an estimated increase per year of 1 extra day, with a standard error of 1 for both analyses. No significant interaction was found between the main effects. In Figure 2, the yearly number of heat stress days averaged across all regions, in total (Days_ta_) and conditional count (Days_c_) is shown together with the increased trend and the standard deviation of the averages.

### Yearly and summer average of THI

The results for the analysis of the evolution of the severity of heat stress accounting for the entire year (average yearly THI) and summer (average summer THI) are shown in Table 3.

**Table 3.** Heat stress severity measured as the least squares means (LSMEANS) of the yearly THI and the summer (June to August) THI per region

Region	Average summer THI ± standard error	Yearly average THI ± standard error
Bergstraße	66.27 ± 0.31	53.06 ± 0.27
Neckar Valley—North	65.39 ± 0.31	52.37 ± 0.27
Neckar Valley—South	65.23 ± 0.31	51.84 ± 0.27
Jagst Valley	63.67 ± 0.31	50.17 ± 0.27
Schussental	64.20 ± 0.31	50.05 ± 0.27
Allgäu—South	64.04 ± 0.31	49.91 ± 0.27
Hohenlohe–Haller–Ebene—Northeast	63.11 ± 0.31	49.49 ± 0.30
Swabian Alps—Northeast	63.06 ± 0.31	49.27 ± 0.27
Iller Valley	63.30 ± 0.31	49.14 ± 0.27
Hohenlohe–Haller–Ebene—Southwest	62.08 ± 0.32	48.86 ± 0.35
Swabian Alps—Center	62.24 ± 0.32	48.48 ± 0.32
Allgäu—North	62.19 ± 0.31	48.30 ± 0.27
Swabian Alps—Southwest	62.55 ± 0.31	48.26 ± 0.28
South Black Forest	62.24 ± 0.32	47.94 ± 0.31
Swabian Alps—Center	62.04 ± 0.31	47.74 ± 0.27
Allgäu—Center	61.56 ± 0.31	47.43 ± 0.30
Swabian Alps—Center	61.03 ± 0.31	47.24 ± 0.27

**Figure 3.**
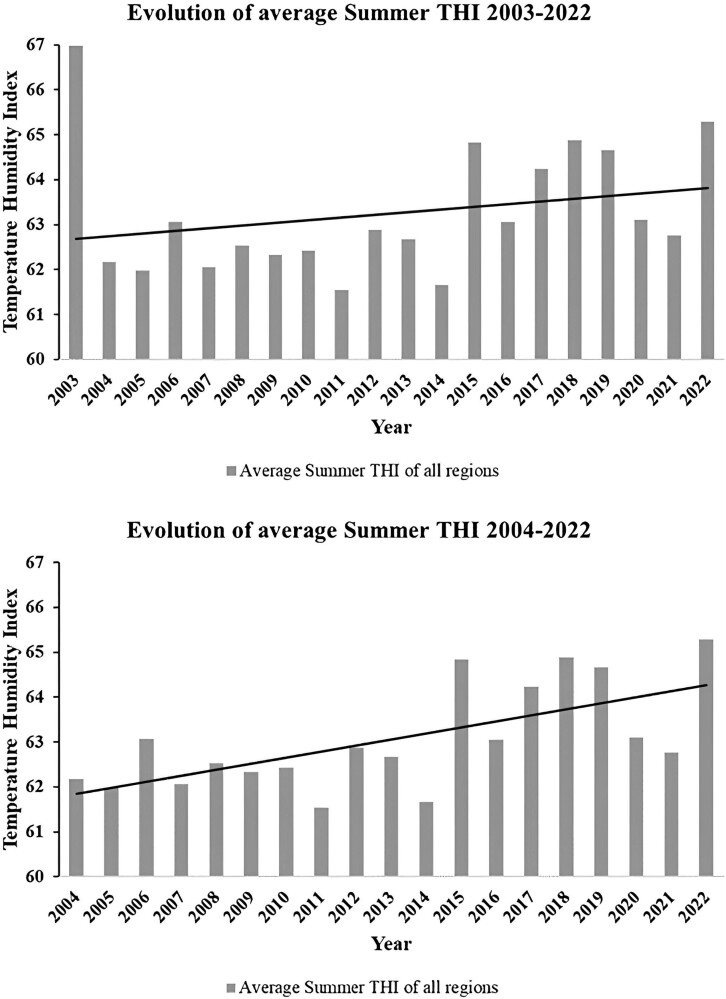
Evolution of the average summer THI across all tested regions for the test period, including the 2003 heat wave (top) and excluding (bottom).

The highest average yearly THI was found in the Bergstraße region with an average of 53.06, followed by the Neckar Valley ranging from 51.84 to 52.37 THI. An average yearly THI of 50.17 was measured in the Jagst Valley and the Schussental regions. The mountains and hills of the Allgäu, Swabian Alps, South Black Forest, and Hohenlohe–Haller–Ebene ranged in THI from 47.24 to 49.91. All variables in the statistical model were significantly different from 0 (α = 0.05), except for the interaction term between the main effects. In Figure 3, the evolution of summer THI across all regions is shown. A severe heat wave in 2003 with increased average THI over the summer months is clearly visible and greatly affects the trend. Bouchama (2004) reported that this heat wave lasted for 9 consecutive days and temperatures reached above 37 °C during the whole period.

**Figure 4.**
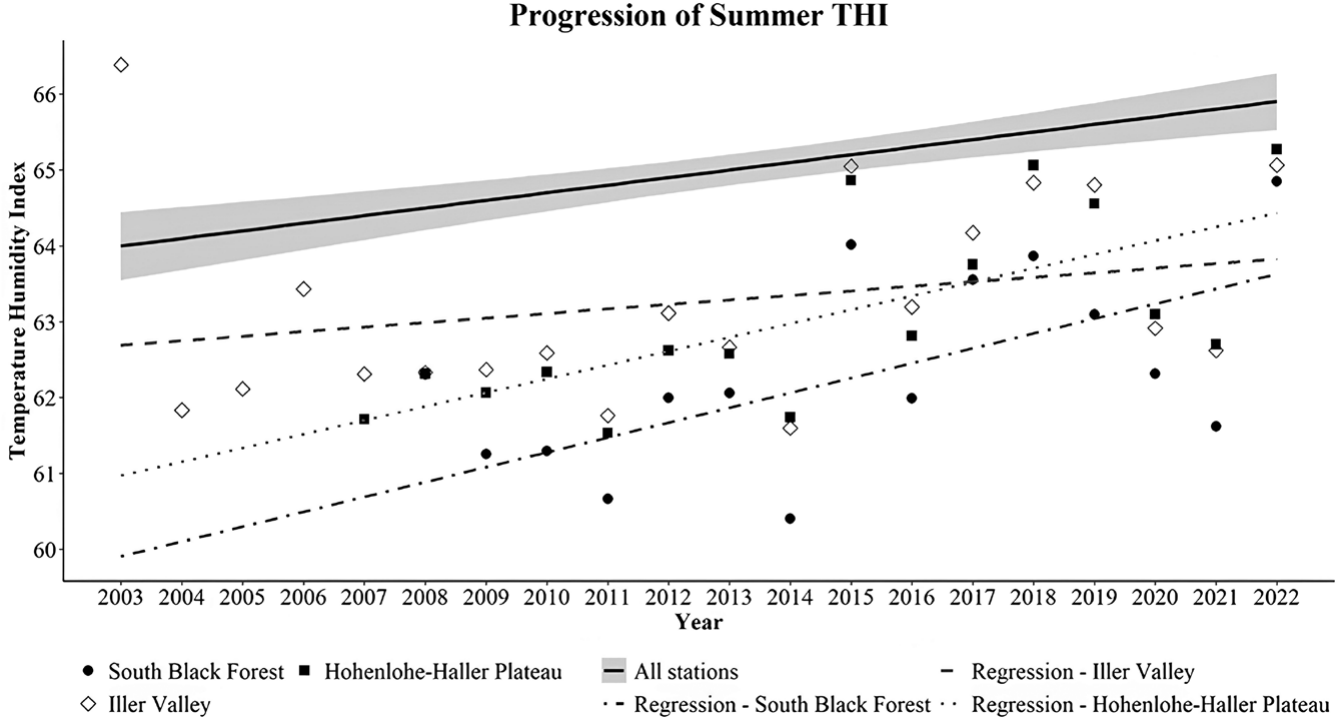
Comparison of the slopes of the regions with differing slopes and the slope for all weather stations. The gray shaded area indicates the confidence band (α = 0.05) of the regression slope of all weather stations. THI of South Black Forest increased from 61.3 to 64.9 in the years 2009 to 2022. THI of the Iller Valley increased from 61.8 to 65.1 in the years 2004 to 2022. THI of the Hohenlohe–Haller Plateau increased from 61.7 to 65.3 in the years 2007 to 2022.

Further, the results of the analysis of the evolution of THI in the summer months yielded a significant (α = 0.05) interaction between the main effects of year and station, as well as month and station. As such, regions were found to be progressing differently in the severity of heat stress during the summer months. Namely, the region of South Black Forest was significantly different (*P* < 0.01) with a slope of 0.1961, so was the region of the Iller Valley (*P* < 0.01) with a slope of 0.0598 and the Hohenlohe–Haller plateau (*P* < 0.001) with a slope of 0.1821. The remaining stations were not significantly different and increased with a common slope of 0.1226. In Figure 4, the comparison between the slopes is presented.

### Classification into heat stress zones—cluster analysis

**Figure 5.**
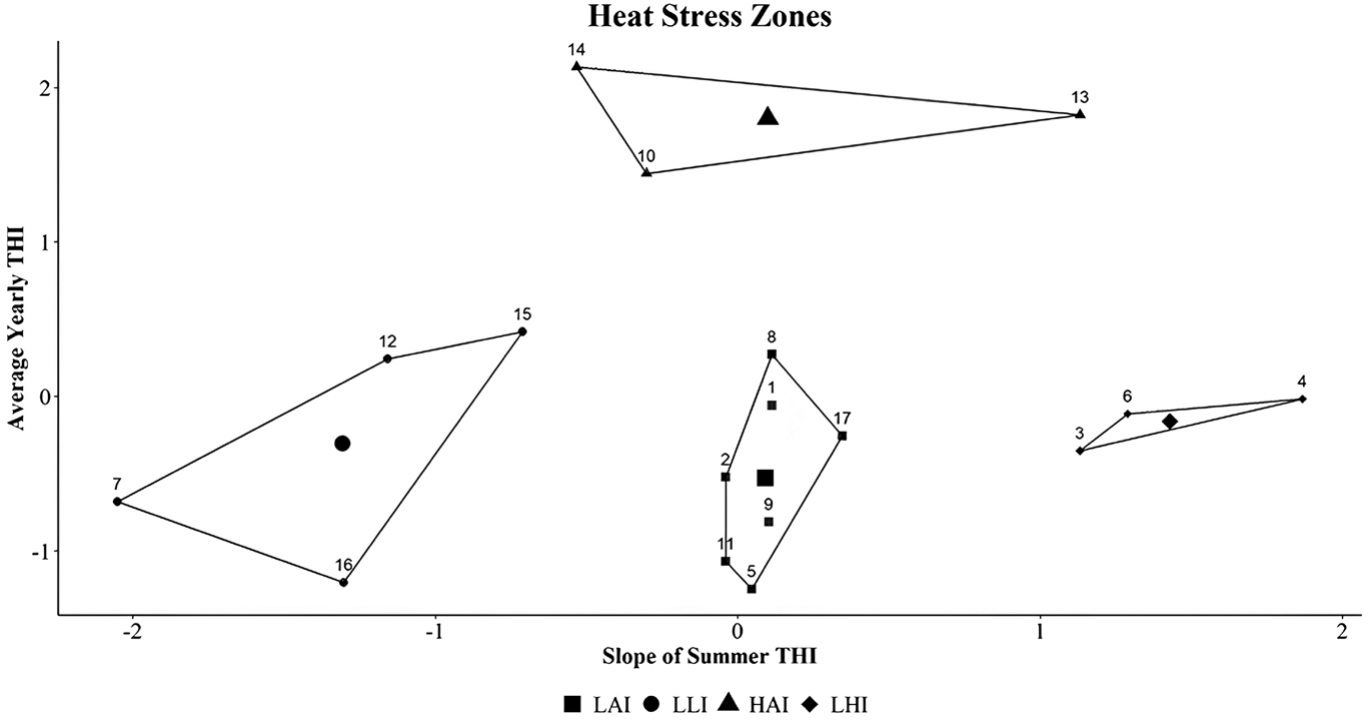
K-Means clusters: LHI, low THI and high slope increase; HAI, high THI and average slope increase; LLI, low THI and low slope increase; LAI, low THI and average slope increase. Values of average yearly THI and slope were scaled for the clustering. The values for slope of summer THI were scaled as follows: −2 = 0.05; −1 = 0.07; 0 = 0.10; 1 = 0.12; 2 = 0.14. The values for average yearly THI were scaled as follows: −1 = 47.7; 0 = 49.4; 1 = 51.1; 2 = 52.8. Cluster position on each axis provided the nomenclature for “low”, “average” and “high” when comparing the clusters. Point numbers refer to stations according to Table 1. Larger symbols within each cluster indicate the average of that same cluster.

**Figure 6.**
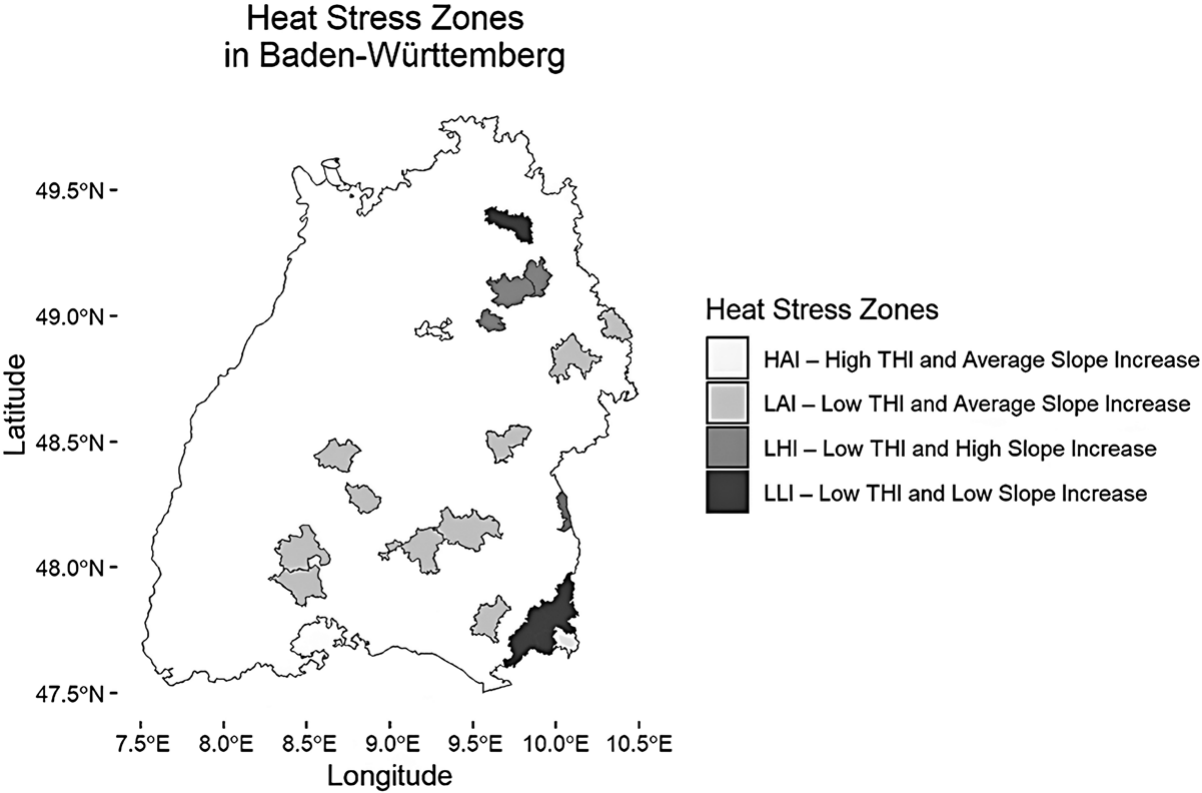
Heat stress zones map of Baden-Württemberg. The highlighted areas correspond to the counties covered by the weather stations in this study. Same color indicates classification into same heat stress zone.

Using the results obtained from the analysis of the average THI for the whole year and the increased slope of the summer THI, the cluster analysis yielded the results seen in Figure 5. The results clearly demarcated the regions similarly to the heat stress period analysis. These were then plotted into a map of Baden-Württemberg for observation in a visual support (Figure 6). These regions are the Allgäu and Jagst Valley with the lowest increase in summer THI per year and low average yearly THI (LLI). The second region comprises the Hohenlohe–Haller–Ebene and the Iller Valley, with the highest increase in summer THI and low average yearly THI (LHI). A third region comprises the Neckar Valley and the Bergstraße, with high average yearly THI and average increase in summer THI per year (HAI). Lastly, the region of the Swabian Alps, South Black Forest, and the Schussental showed an average increase in summer THI per year and a low average yearly THI (LAI).

### Heat stress effect on milk production

**Figure 7.**
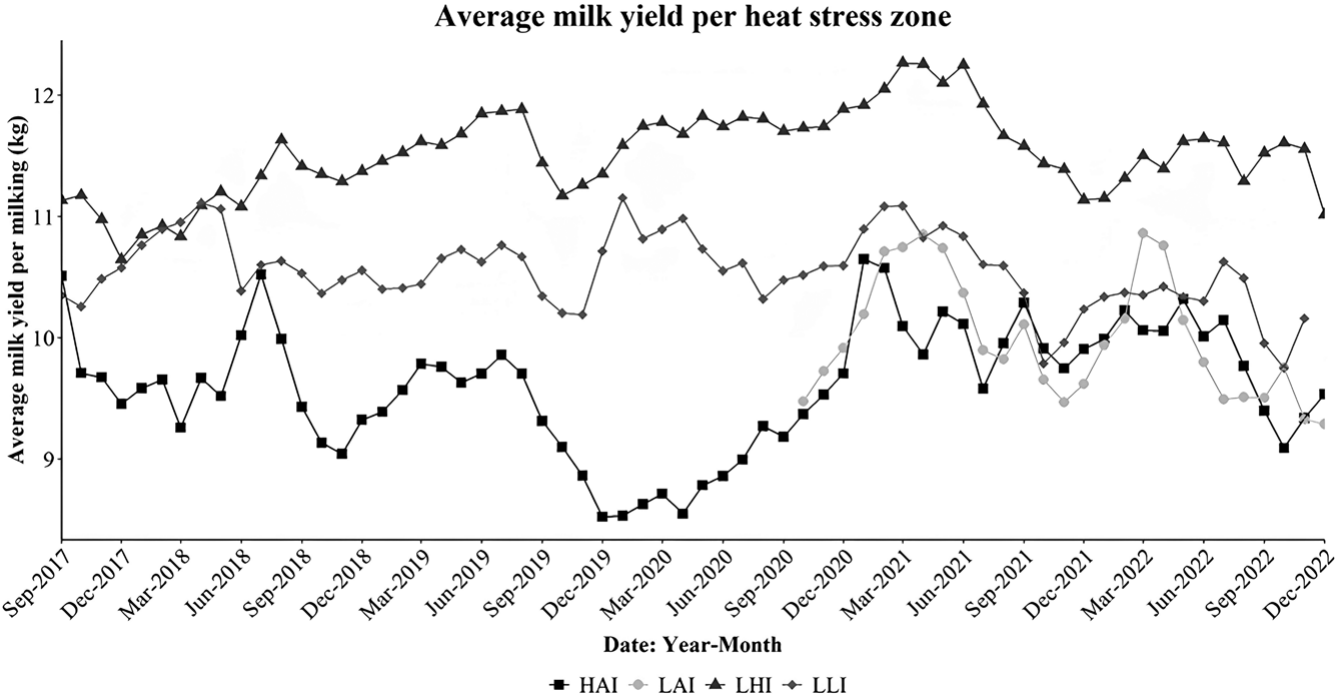
Progression of the average milk yield per milking (kg) per heat stress zone in the period between September 2017 and December 2022. Milking yields data for zone LAI was only available starting October 2020.

The results of the effects of heat stress on milk production in the temperate regions are shown in Table 4. The average, lowest and highest milk yield per milking for each of the heat stress zones are shown. Low THI, in zones LHI, LLI, and LAI, was found to yield the highest milk yield per milking at 11.52, 10.55, and 9.99 kg, respectively. The highest average THI, in zone HAI, yielded the lowest average milk yield per milking at 9.60 kg. The analysis of variance for the effects of heat stress on milk production in the temperate regions yielded a significant effect (*P* < 0.001) of heat stress zone on the average milk yield per milking. Similarly, the interaction between heat stress zone and date was also found to be significant (*P* < 0.01), that is, the heat stress zones progress differently through time regarding the average milk yield per milking. Variation within the LAI heat stress zone means it intersects the curves for the zones HAI and LLI, as such its positioning as higher or lower than any of those is dependent on the measuring date (Figure 7). As measurements for the farms located in this zone were only available from May 2020, test of significant difference was not possible. Heat stress zones LLI, HAI, and LHI were found to be significantly different (*P* < 0.01) from each other.

**Table 4.** Average, lowest and highest milk yield per milking for each heat stress zone across the test period, September 2017 to December 2022. Milking yields data for zone LAI was only available starting May 2020. LHI, low THI and high slope increase; HAI, high THI and average slope increase; LLI, low THI and low slope increase; LAI, low THI and average slope increase; SD, standard deviation

Milk yield per milking (kg)	LAI	LLI	HAI	LHI
Average ± SD	9.99 ± 0.50	10.55 ± 0.31	9.60 ± 0.52	11.52 ± 0.35
Lowest	9.29	9.75	8.53	10.65
Highest	10.86	11.15	10.65	12.27

## Discussion

### Heat stress duration and severity

The current study set out to find out whether heat stress is a growing problem for cow milk production in the temperate regions or not. The results on the trends of increasing average summer THI and on the steady increase of the length of the heat stress period indicate the affirmative, albeit the trends are muted by the cyclical nature of climate. The study also found that heat stress duration and severity varied in different locations. It can be expected that further North temperature is lower, however, THI accounts for ambient temperature and relative humidity, therefore, lower temperatures do not necessarily result in less heat stress if relative humidity is high. In this study, independently of the geophysical characteristics of the different locations, the length of the heat stress period in days was found to be increasing equally in all locations at the rate of one extra day per year, as determined by the slope estimate of the linear mixed model, and as corroborated by existing research on heat stress perception by humans (Matzarakis and Mayer, 1997). This represents an increase of 16% to 33% of the number of heat stress days per year over a period of 20 yr. By comparison, Lorenz et al. (2019) found an increase of up to 300% of the number of heat stress days in a year for central Europe over the last 20 yr, and an increase of summer heat stress severity of 50% over the average. The length of the heat stress period observed in the recent years was found to be up to 120 d, 4 mo a year, of which 107 d were within periods of 5 or more consecutive days, as opposed to the expected 3 mo of summer. Similar climate research has found an increase in up to 50% of the number of warm days for the months of March and May, and up to 30% in the Autumn months (European Climate Change Service, 2023). The shortest duration of heat stress period was 64 d, of which 49 d were within periods of 5 or more consecutive days, and the difference between the different regions was not related to their location North-South, but rather to their geophysical properties. These findings suggest that variation induced by climate change may be independent of small to negligible latitude changes and affects the duration of the heat stress period, that is, the increase in the number of days per year when heat stress occurs. Different heat stress zones were found that include a variety of environments of different temperate regions, where low to high rates of increase of average summer THI were found and low to high heat stress severity was detected. The interaction of climate change with environments, even those in close proximity, appeared to produce great variety in heat stress patterns, suggesting a close interaction with microclimates. Herbut et al. (2018) mention the necessity to predict heat stress by observing microclimates, as all the factors accounted for in different heat stress measuring equations are those determining microclimates. Other studies specify these as wind, precipitation, solar exposure, and topography among others (Buffington et al., 1981; Da Silva et al., 2010). In this study, however, wind, precipitation, and solar exposure were not accounted for as all animals were permanently stabled. In other production systems, those parameters could be necessary.

The occurrence of a strong heat wave in 2003, the hottest summer in Europe since recordings existed (Luterbacher et al., 2004), greatly changed the rate of increase in summer THI in all identified heat stress zones. While removing the heat wave THI values from the models would greatly increase the rate and drastically worsen the scenarios proposed, heat waves and their frequency are commonly associated with climate change and therefore worsening heat stress (Miller et al., 2021). Therefore, that heat wave in 2003, as well as smaller ones that occurred in 2015, and 2018 were included in the analysis and resulting models. Of note was that the heat stress period duration in days was unaffected by the heat waves, not affecting the yearly increase that always remained at an average of one extra day per year. As such, heat waves were found to increase the severity of heat stress while they happen (Figure 3), but they do not extend the heat stress period beyond the already existing tendency.

### Geophysical characteristics of heat stress zones

The current study has also demonstrated the importance of microclimates to heat stress. Within the environments present in the test region, most severe heat stress was found on the exposed foothills of the South face of mountains and in low-altitude river valleys. These environments showed an average yearly increase of THI in the summer months and registered the longest period of heat stress each year. Relative humidity in this environment was found to be high, while the main detected wind directions were North over the mountains, South over plains, and the prevailing winds from West (Deutscher Wetterdienst, 2013). This location in south face of mountain ranges and opposing winds suggests a microclimate with trapped humidity and prolonged exposure to solar radiation increasing ambient temperature. The combination of high relative humidity and ambient temperatures would necessarily result in higher THI.

Mountainous environments yielded results grouped into one heat stress zone with average severity of THI and average summer THI increase, as per the cluster analysis. Heat stress period in days per year showed great variance, most likely due to the many different levels of exposure to the sun, varying altitude, and its effects on relative humidity. As such, the pattern found was that the increase in average THI accompanies the expectable effects of climate change, that is the slope of increase is only average. However, absolute values of THI already present are dependent on the exact location and weather exposure of each mountain face. Within the microclimates of the mountainous areas, the most severe heat stress was found in the North-East faces where humidity accumulates, and sun exposure occurs for at least a part of the day. The least severe heat stress occurred in the high exposed altitudes where wind and lower temperatures provided good conditions for cattle. These varied results suggest that in mountain ranges, a comprehensive analysis of heat stress requires contiguous measurements over the mountain range, as varying topography reduces how representative any area can be relative to their neighboring areas.

Hilly areas and river valleys within hills shared a similar characteristic, that is a low heat stress severity and low summer THI increase. Given the proximity of the Lake of Constance to the hills in this study, it is possible that high relative humidity contributed to approximate these 2 different environments in their heat stress characteristics. These results indicate that in average-altitude microclimates with high relative humidity and low wind speed, average THI will be low and the trend is for a slow yearly increase. This suggests that either a constant removal of humid air reduces the occurrence of heat stress, or lower temperatures due to altitude increase relative humidity to the point of precipitation. A combination of both throughout the year is also a potential cause for a better environment for dairy cows. Furthermore, the higher weight of ambient temperature in THI calculations compared to relative humidity could also explain the low average THI in these microclimates. This would be similar to other dairy production areas, such as the high-altitude dairy farming in the Alps (Renna et al., 2010), and to the reasoning behind common mitigation strategies in coastal areas, where humidity removal is the main focus (Matzarakis and Mayer, 1997; Flamenbaum and Galon, 2010; Luo and Lau, 2018).

The plateaus, flatlands at higher altitudes surrounded by mountain ranges, showed the lowest heat stress severity but also showed the highest increase in summer THI. Environments with these geophysical characteristics, such as flatlands at higher altitude, are common within the temperate regions and, depending on the altitude, range from desertic tundra (on the extreme example: the Tibetan Plateau and Andine desert), to low altitude rocky formations (just above 200 m). As extensively explained by Ruddiman and Kutzbach (1991), the common element to these microclimates would be low relative humidity despite the presence of rivers, hence the low THI, but also a much lesser buffer capacity for increased ambient temperature in the warm months. Low relative humidity, is mostly due to precipitation occurring in the surrounding mountains, leaving dry wind to circulate over the plateau under prolonged exposure to the sun.

### Microclimates

Results in the current study confirmed the association between milk yield and heat stress. The effect of heat stress on milk production is mainly through direct and indirect influences. The direct oppressive effect of heat load affects the physiological processes in the animal while the indirect effects are through reduced feed and water intake. Previous studies have demonstrated the reduction in milk production with increased temperatures and heat wave frequency (e.g., Silanikove and Koluman (Darcan) 2015; Guzmán-Luna et al., 2022), Further, many studies have addressed issues relating heat stress with reduced feed quality (Spiers et al., 2004; Renna et al., 2010), which combined with lower feed intake (Ayemele et al., 2021) results in a drastic reduction of milk production. Few studies, however, have analyzed the effect microclimates have on heat stress and how microclimates affect any estimations and predictions of how heat stress will behave in the future under increased pressure from climate change. Two such studies on dairy cows, Herbut et al. (2018) and Da Silva et al. (2010), base the importance of microclimates on much older studies Buffington et al., 1981). More recent studies relating heat stress and microclimate have been performed in urban environments, analyzing human perception of heat stress, the detrimental effects on physical and mental health as well as mitigation strategies (Matzarakis and Mayer, 1997; Harlan et al., 2006). Nevertheless, while tolerance thresholds differ greatly from humans to animals, the authors concluded that trapped humidity, heat absorbed by structures and excessive sun exposure as well as excessive shielding from the wind all contribute to increasing heat stress. These results can be extrapolated to the topography of the environments in this study, where South and East Mountain faces, sheltered from some wind directions trap moisture, and have prolonged sun exposure. Plateau environments are sheltered from substantial humidity by surrounding mountains, however in the summer, with increased temperature, the water carry capacity of the air increases, greatly reducing relative humidity to a situation more akin to those found in the subtropics and arid regions as discussed by Bohmanova et al. (2007) and Avendano-Reyes (2012). Mitigation strategies for heat stress in drier areas will differ from those in humid areas. Drier areas provide the animal with more opportunity to balance body temperature through transpiration and panting, while humid areas restrict the use of this ability. This leads to earlier occurrence of heat stress symptoms such as lower feed intake and disease festering. Microclimates in hills and areas exposed to winds with enough altitude to reduce temperatures and balance relative humidity appear to present the best environment of those tested for dairy production and slow rates of THI increase. Research and management choices related to the effect of heat stress on dairy cattle farms should, in the future, more often account for the effect of microclimate rather than overarching climate tendencies.

## Conclusions

This study has demonstrated that heat stress in the temperate regions is a worsening issue. The number of heat stress days per year increased at a rate of 1 extra day per year and reached up to 120 d/yr in some regions. The severity of heat stress within the temperate regions was found to depend on what microclimate characteristics each location is subjected to, and to progress at different rates in different regions. A permanent effect on the milk production level could already be detected. Distinguishing regions was possible by comparing each individual heat stress severity level with the rate of increase of that same severity, demarcating different heat stress zones. This suggests that the temperate regions will sustain decreases in milk yield and, potentially, in animal health. Management strategies and animal breeding for better performance and/or resilience taking each local microclimate into account can provide the best response to those scenarios. The increase in heat stress occurred in all regions and varied only in degree in relation to each microclimate.

## Abbreviations

AMS: automated milking systems
Daysta: total annual count of days with Temperature–Humidity Index ≥ 60
Daysc: consecutive count of at least 5 d with Temperature–Humidity Index ≥ 60
RH: relative humidity
T: ambient temperature
THI: Temperature–Humidity Index
